# Explaining outcomes in major system change: a qualitative study of implementing centralised acute stroke services in two large metropolitan regions in England

**DOI:** 10.1186/s13012-016-0445-z

**Published:** 2016-06-03

**Authors:** Naomi J. Fulop, Angus I. G. Ramsay, Catherine Perry, Ruth J. Boaden, Christopher McKevitt, Anthony G. Rudd, Simon J. Turner, Pippa J. Tyrrell, Charles D. A. Wolfe, Stephen Morris

**Affiliations:** 1 Department of Applied Health Research, University College London, London, UK; 2 Alliance Manchester Business School, University of Manchester, Manchester, UK; 3 Department of Primary Care and Public Health Sciences, Division of Health and Social Care Research, Faculty of Life Sciences and Medicine, King’s College London, London, UK; 4 National Institute of Health Research Comprehensive Biomedical Research Centre, Guy’s and St Thomas’ NHS Foundation Trust and King’s College London, London, UK; 5 Guy’s and St Thomas’ NHS Foundation Trust, St Thomas’ Hospital, London, UK; 6 Stroke and Vascular Centre, University of Manchester, Manchester Academic Health Science Centre, Salford Royal Hospitals NHS Foundation Trust, Salford, UK; 7 National Institute of Health Research Collaboration for Leadership in Applied Health Research and Care (CLAHRC) South London, London, UK

**Keywords:** Implementation approaches, Implementation outcomes, Evaluation, Stroke care, Centralisation of healthcare

## Abstract

**Background:**

Implementing major system change in healthcare is not well understood. This gap may be addressed by analysing change in terms of interrelated components identified in the implementation literature, including decision to change, intervention selection, implementation approaches, implementation outcomes, and intervention outcomes.

**Methods:**

We conducted a qualitative study of two cases of major system change: the centralisation of acute stroke services in Manchester and London, which were associated with significantly different implementation outcomes (fidelity to referral pathway) and intervention outcomes (provision of evidence-based care, patient mortality). We interviewed stakeholders at national, pan-regional, and service-levels (*n* = 125) and analysed 653 documents. Using a framework developed for this study from the implementation science literature, we examined factors influencing implementation approaches; how these approaches interacted with the models selected to influence implementation outcomes; and their relationship to intervention outcomes.

**Results:**

London and Manchester’s differing implementation outcomes were influenced by the different service models selected and implementation approaches used. Fidelity to the referral pathway was higher in London, where a ‘simpler’, more inclusive model was used, implemented with a ‘big bang’ launch and ‘hands-on’ facilitation by stroke clinical networks. In contrast, a phased approach of a more complex pathway was used in Manchester, and the network acted more as a platform to share learning. Service development occurred more uniformly in London, where service specifications were linked to financial incentives, and achieving standards was a condition of service launch, in contrast to Manchester. ‘Hands-on’ network facilitation, in the form of dedicated project management support, contributed to achievement of these standards in London; such facilitation processes were less evident in Manchester.

**Conclusions:**

Using acute stroke service centralisation in London and Manchester as an example, interaction between model selected and implementation approaches significantly influenced fidelity to the model. The contrasting implementation outcomes may have affected differences in provision of evidence-based care and patient mortality. The framework used in this analysis may support planning and evaluating major system changes, but would benefit from application in different healthcare contexts.

**Electronic supplementary material:**

The online version of this article (doi:10.1186/s13012-016-0445-z) contains supplementary material, which is available to authorized users.

## Background

The field of implementation science articulates the need for a nuanced approach when evaluating the outcomes of change. An important distinction is drawn between ‘implementation outcomes’, i.e. the adoption of, fidelity to, and sustainability of a given intervention [1–9], and ‘intervention outcomes’, for example, changes in provision of care or patient outcomes [4]. This enables study of factors that influence implementation (including the nature of the intervention and how its implementation is facilitated), and the potential relationships between these and intervention outcomes [4, 6], allowing insights into the ‘black box’ of implementation.

Understanding how evidence-based practice is implemented in complex settings such as healthcare is enhanced when its various components are considered (decision to change, intervention selection, planning and implementation of change, and outcomes) [2, 5, 6, 10]. The value of theory, as represented through conceptual frameworks, is recognised as benefitting the design, application, and understanding of implementation approaches [2, 11–13]. Such frameworks provide, firstly, an analysis of how contextual factors, such as national policy or a ‘burning platform’, can influence the decision to change, and the type of intervention that is implemented [8, 11, 13]. Second, how characteristics of the intervention implemented (e.g. a new service model), such as its complexity or its compatibility with local context, might influence the outcomes of implementation [3, 7, 9, 10, 13]. Third, how the implementation approaches employed, i.e. how change is facilitated, managed, and led, can influence implementation outcomes [1, 2, 10, 11, 13, 14].

However, research exploring the relationships between implementation approaches, implementation outcomes, and intervention outcomes remains limited [4]. To address this gap, we present a mixed methods evaluation of major system change of acute stroke care; these changes took place in two large metropolitan regions in England, London, and Greater Manchester (hereafter ‘Manchester’), which had significantly different intervention outcomes [15].

### Implementing major system change in healthcare settings

Major system change in healthcare is seen as having the potential to increase the provision of evidence-based care and improve clinical outcomes [14]. It therefore represents an important area for implementation research. Major system change involves reorganisation of services (sometimes termed ‘reconfiguration’ [16]), at regional level, and may include significant alterations to a care pathway. One such change is service centralisation, whereby service provision across a given region is concentrated in a reduced number of hospitals [17–22]. It may involve many stakeholders across multiple organisations, and—when implemented successfully—is hypothesised to optimise the balance between quality of care, access, workforce capacity and cost [14]. The impact of centralisation on outcomes has been demonstrated in several specialist healthcare settings, including trauma [23–25], cardiac surgery [26], neonatal intensive care [27], and acute stroke care [28, 29]. However, evidence on how changes of this scale are implemented, and the relationship between implementation approaches and the impact of changes on quality of care and costs, remains limited [14]. For example, a review of the evidence of ‘successful’ and ‘less successful’ major system changes in healthcare settings defined ‘success’ in relation to implementation outcomes rather than intervention outcomes [8].

### Developing a framework to analyse major system change

**Fig. 1:**
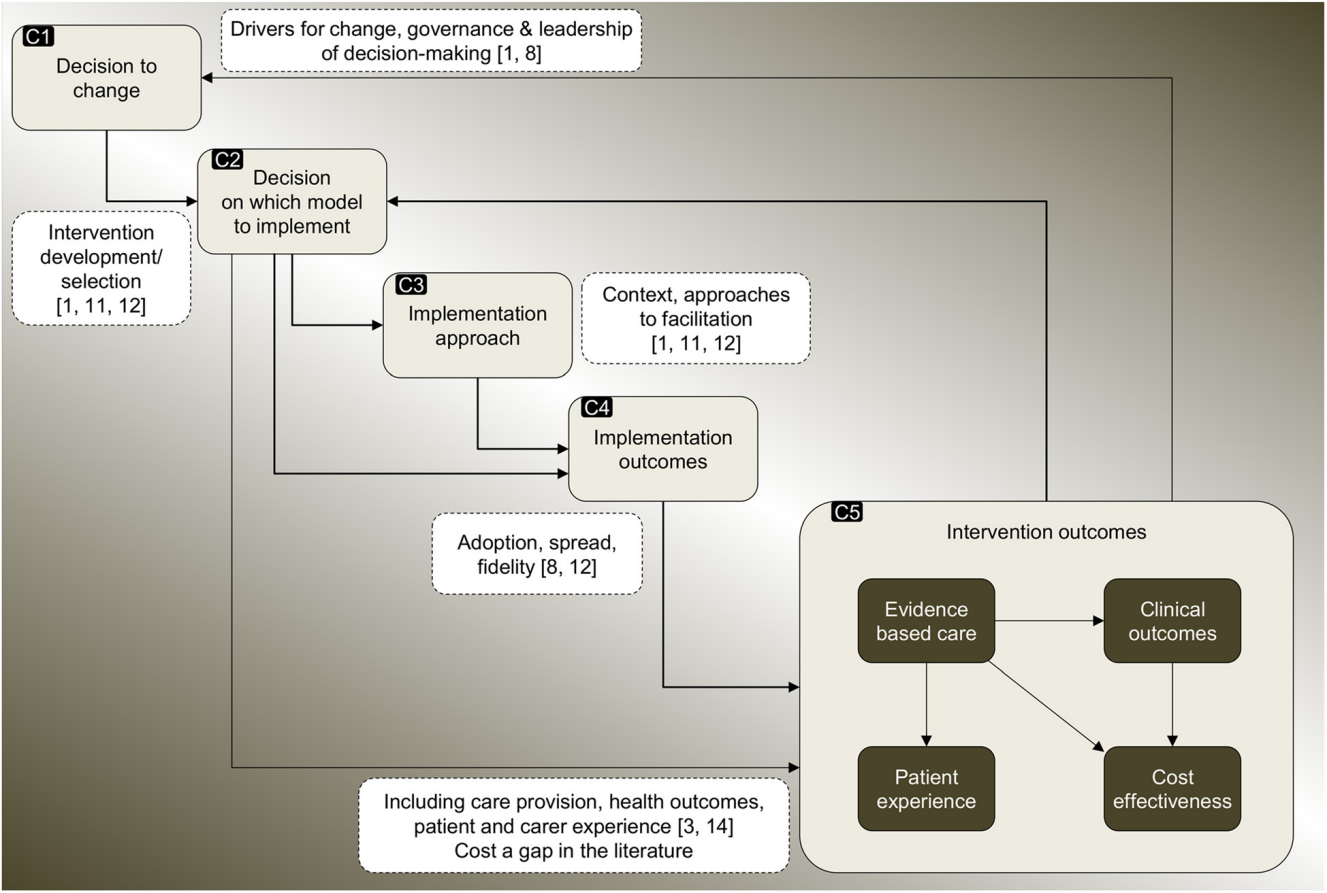
Conceptual framework: key components of major system change

Drawing on the literature on implementation and major system change described above, we have developed a schematic framework that identifies key components of major system change, and how they might interact (Fig. 1). The framework distinguishes between *implementation* outcomes and *intervention* outcomes.

The decision to change, e.g. the drivers for change, governance, and leadership of the decision-making process (component 1 (C1), Fig. 1) may influence the nature of the model (i.e. the intervention) that is implemented (C2) [8, 10]. Through processes of adaptation, both contextual factors (e.g. managerial capacity to lead change) and the model selected (e.g. the scale of change required) may influence the implementation approaches used (e.g. the degree to which local staff may require hands-on support in managing change) (C3) [1, 9]. Through both its complexity and its compatibility with the context of its introduction, the model selected may also influence implementation outcomes, in terms of uptake and fidelity [4, 7]. The model may influence intervention outcomes directly, though it is important that the extent to which the effects of the model are mediated through the process of implementation be considered [4]. Implementation approaches, such as how change is facilitated and local staff are supported (C3), have potential to influence implementation outcomes (C4) [1, 8]. Implementation outcomes (C4) are likely to influence overall intervention outcomes, including provision of evidence-based care, clinical outcomes, patient and carer experience, and cost-effectiveness (C5) [4]. Finally, assessment of implementation outcomes may prompt a decision to change again and implement amended or alternative models [9]. The relationships between these components are unlikely to be linear; some (e.g. C1-3) may occur simultaneously, and some components may be bypassed, e.g. model characteristics (C2) may influence implementation outcomes (C4) directly.

### Major system change in Manchester and London acute stroke services

**Fig. 2:**
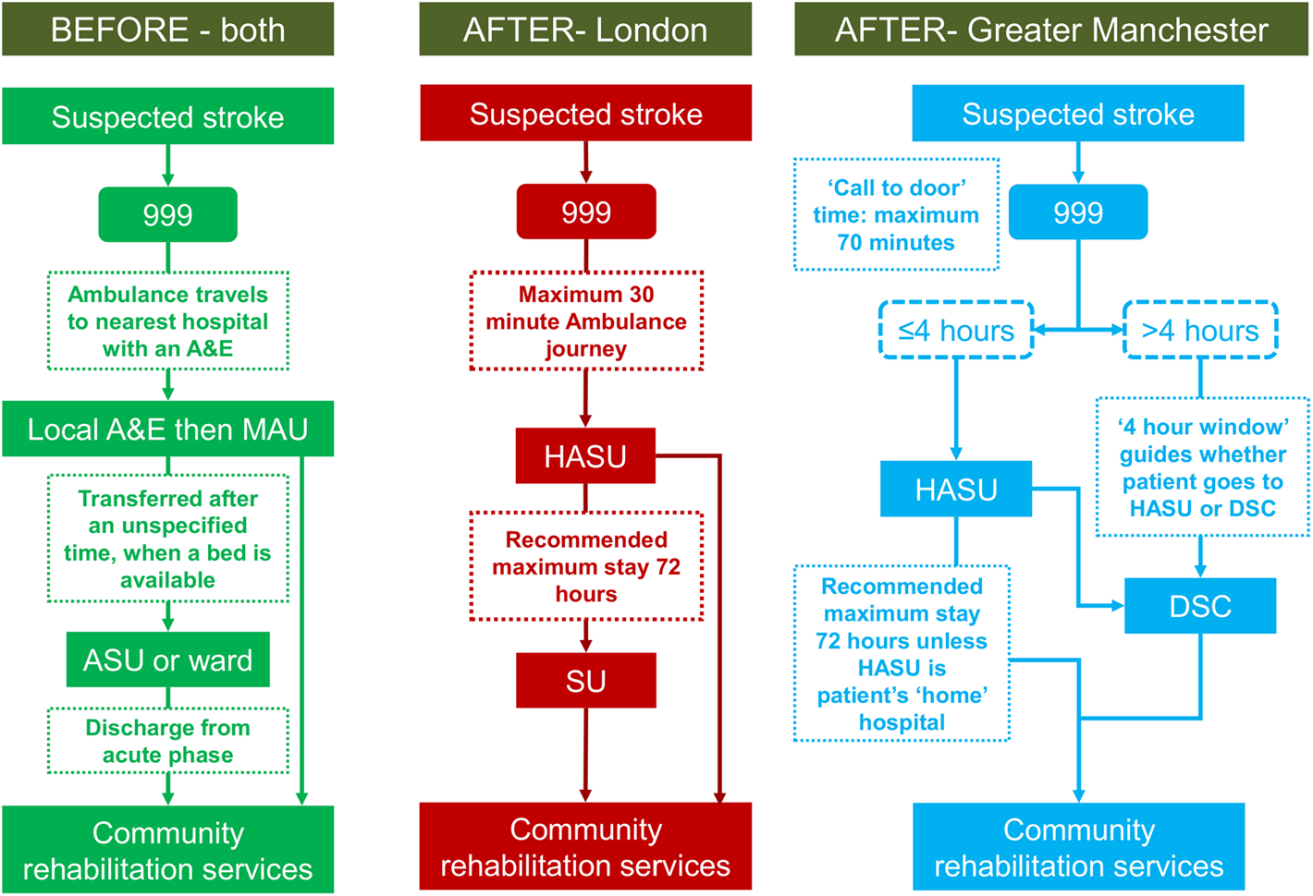
Overview of major system changes in London and Manchester stroke services. *A&E* accident and emergency ward, *MAU* medical assessment unit, *ASU* acute stroke unit, *HASU* hyperacute stroke unit, *SU* stroke unit, *DSC* district stroke centre

In 2010, London and Manchester implemented a major system change of their acute stroke services; these were reorganised in order to improve rapid access to evidence-based care, including assessment by specialist stroke clinicians, rapid brain scanning, and thrombolysis where appropriate (a time-limited ‘clot-busting’ treatment that needs to be administered within 4 h of symptom onset [30, 31]). The changes to service models are summarised in Fig. 2.

In each region, a small number of hyperacute stroke units (HASUs) were designated to deliver these evidence-based care processes. In addition, in London, 24 stroke units (SUs) were designated to provide acute rehabilitation to patients until they were ready to return to the community. In Manchester, 10 district stroke centres (DSCs) were designated to provide all aspects of acute stroke care required beyond the first 4 h. Referral pathways differed in terms of ‘inclusivity’; whereas all patients in London were eligible for treatment in a HASU (the ‘24 h pathway’), in Manchester only patients arriving at hospital within 4 h of symptoms developing (in order to facilitate administration of thrombolysis) were eligible, with patients presenting later transferred to their nearest DSC (the ‘4 h pathway’). Further, while stroke services in five hospitals were closed in London as part of the changes, no services closed in Manchester [15, 32]. These significant differences in the type of models implemented in the two regions reflect the limited evidence at the time on optimal service models for providing evidence-based care [32]. Stroke clinical networks (hereafter referred to as ‘networks’) played an important role in the changes. Networks were set up following the national stroke strategy, and brought together representatives of all relevant stakeholder groups under a central leadership team, in order to ‘review and organise delivery of stroke services across the care pathway’ [33] .

**Fig. 3:**
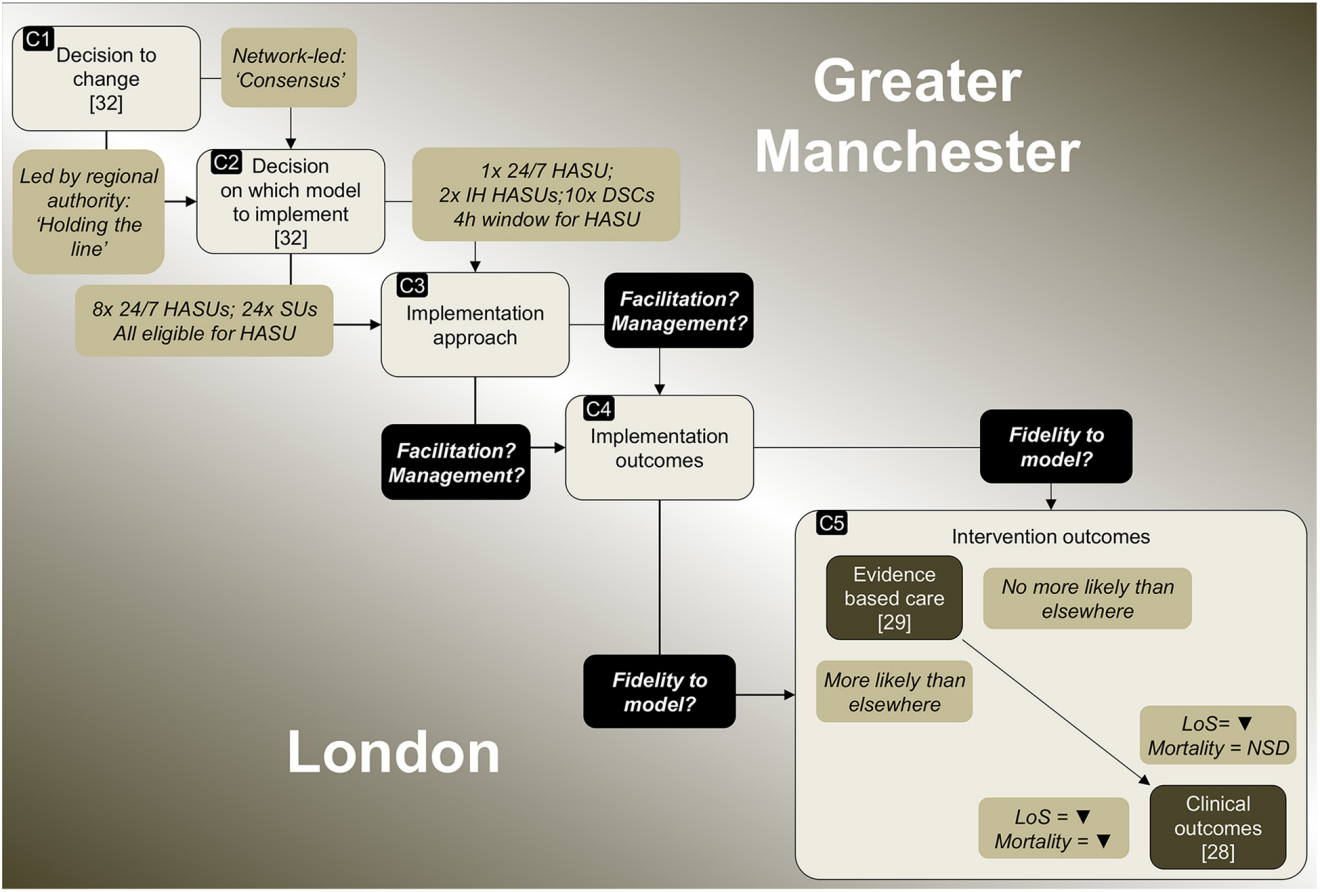
Current findings on major system changes in London and Manchester stroke services. *HASU* hyperacute stroke unit, *DSC* district stroke centre, *IH* in-hours, *LoS* length of hospital stay, *NSD* no significant difference

To date, our study of these major system changes has allowed us to populate certain components in our framework (Fig. 3). We have established that the drivers for major system change in both regions included national policy and local awareness of unacceptable variations in and overall quality of acute stroke care provision [32]. We have also established important differences in how the decision to change was led and governed, how local resistance was managed (C1, Fig. 3) [32], and how these influenced the models selected (C2) [32]. Secondly, we have established that the changes in London and Manchester were associated with different intervention outcomes; London patients were significantly more likely to receive evidence-based care than patients in Manchester (C5) [29]; and only London was associated with significantly greater reduction in stroke patient mortality compared to other urban regions of England (C5) [28].

In this paper, we present a comparative study of these two major system changes, examining the relationships between implementation approaches employed and the implementation outcomes (C3 and C4, Fig. 3). We address how implementation approaches and implementation outcomes were influenced by differences in the model selected (C2, Fig. 3) and how they influenced the differing intervention outcomes (C5, Fig. 3). Through this analysis, we will contribute to understanding of implementation of major system change in terms of the relationships between the models selected and implementation approaches applied, and how these each may influence implementation outcomes and intervention outcomes (C3–C5, Fig. 3) [34].

## Methods

### Setting

The changes took place in Manchester and London (populations 2.68 and 8.17 million, respectively [35]). Implementation took place in Manchester between December 2008 and April 2010, and between October 2009 and July 2010 in London.

### Study design

We focused on Manchester and London as the only examples of changes of this kind being implemented at such a scale at the time [15]. Qualitative fieldwork, combining documentary analysis and interviews, was undertaken at ‘governance’ and ‘service’ levels to compare the implementation of the changes in the two regions. At governance level, interviewees were purposively sampled to obtain national and pan-regional perspectives on planning and implementation of the centralisations. At service-level, a number of stroke services were purposively sampled to capture the range of experiences of the changes. In Manchester, we sampled: the sole 24/7 HASU; one of the two in-hours HASUs; one of the 11 DSCs; and the ambulance service. In London, we sampled two of eight HASUs, on the basis of both performance on the pre-designation service assessment and location (because both were factors considered in the final designation of HASUs); two of the 24 SUs from different areas; the ambulance service; and one of the five services that were decommissioned. Interviews were conducted with clinicians and managers within these services (see Table 1) [15]. We sought to obtain all relevant policy documents at national and regional level and documents related to planning and implementation of changes at regional and service level (see Table 1).

**Table 1:** Summary of data analysed

Data	Sources	London	Manchester	Total
Documents	Project plans, consultation documents, impact assessments, external reviews, designation criteria, service protocols, meeting minutes	386	267	653
National level interviews	Politicians; clinical leaders with national remit	–	–	4
Pan-regional level interviews (governance level)	Planners and leaders of changes, including programme managers, committee chairs, commissioners, system managers, network representatives, and patient organisations	25	16	41
Service level interviews	Clinicians, service managers, and senior managers:			
	Manchester: 24/7 HASU	–	11	11
	Manchester: in-hours HASU	–	10	10
	Manchester: post-4 h DSC	–	11	11
	London: HASU, North London, high score	11	–	11
	London: HASU, South London, low score	12	–	12
	London: SU, North London	8	–	8
	London: SU, South London	8	–	8
	London: decommissioned service	4	–	4
	Ambulance	2	3	5
Service level total		45	35	80
Total interviews		70	51	125

### Data

We combined analyses of semi-structured stakeholder interviews and documents. We conducted 125 semi-structured interviews with stakeholders at governance (*N* = 45) and service (*N* = 80) levels (Table 1) over the period April 2012 to December 2013.

Interviews at governance level covered background to the centralisations (including drivers for change); governance; developing the proposal for change; agreeing the model; implementing changes; impact of centralisation; and reflections on the changes (see Additional file 1). Interviews at service level covered background to changes; processes of service development; impact of centralisation; and reflections on changes (see Additional file 2). In addition, 653 documents were collected from governance and service levels (Table 1).

### Participant recruitment and data collection

Potential interviewees were contacted via e-mail or telephone. Interviews were conducted only with fully informed, written consent and were audio-recorded and professionally transcribed. All documents analysed were either in the public domain, or obtained from local change leaders and service leads.

### Analysis

We compared the London and Manchester changes in terms of the implementation approaches employed and the implementation outcomes. Findings were considered in relation to our previously published findings, i.e. the different models implemented [32], and their differing impact on intervention outcomes (likelihood of patients receiving evidence-based care [29], and patient mortality [28]).

Data analysis from interviews and documents combined inductive and deductive approaches [36], as themes were drawn from our framework (Fig. 1) and emerged from the empirical data. Documents were analysed to identify various aspects of the changes, including drivers, key events and activities, and overarching chronology. Interviews were analysed to draw out similar information, and to understand why and in what ways aspects of implementation were influential, in order to compare the two regions. Analysis took place in three phases, building on the narrative summaries and timelines of the changes developed from documentary analysis used in a previous analysis [32]. In phase one, service-level narrative summaries were developed, using the constant comparative method [37], from documentary evidence and initial readings of interviews. These were developed separately for the changes in London and Manchester (by AIGR and CP), and covered a number of cross-cutting themes: service-level context; service development processes (including thrombolysis and repatriation protocols, recruiting, and training staff); launching new services; and perceived impact of changes. In phase two, we used the overall timelines and summaries and service-level summaries to identify key tasks in implementing the models in each region, and contrasts in how these tasks were accomplished. In phase three, a subgroup of the authors (CP, AIGR, SM, and NJF) applied the framework (Fig. 1) to a cross-region analysis that sought to test explanations of the differing implementation outcomes identified in previously published quantitative analyses. This phase drew on further thematic analysis of interview and documentary data to identify factors influencing the contrasting implementation approaches, and how the approaches may have influenced the resultant implementation outcomes.

To enhance reliability, emerging findings from each phase were shared and discussed regularly with other co-authors until an agreement was reached. To enhance validity, an interim version of this analysis was shared with people who had been involved in the planning and implementation of the changes in London and Manchester (some of whom we had interviewed for this study).

#### Ethical approval

This study received ethical approval in September 2011 from the London East NHS Research Ethics Committee (Ref 11/LO/1396).

## Results

**Fig. 4:**
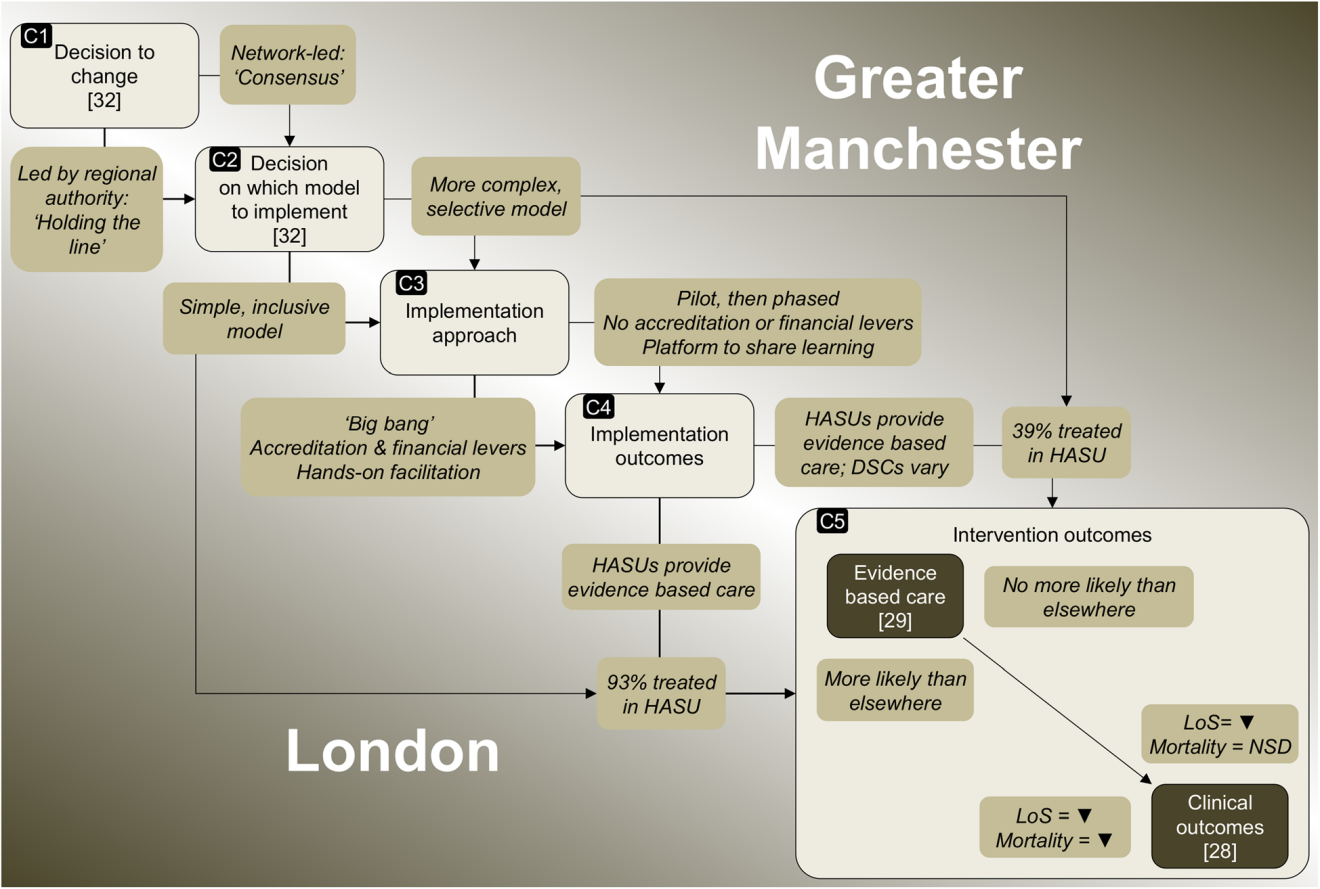
Findings in relation to major system change in London and Manchester stroke services. *HASU* hyperacute stroke unit, *DSC* district stroke centre, *LoS* length of hospital stay, *NSD* no significant difference

We present our findings in three sections: factors influencing implementation approaches; factors influencing implementation outcomes; and understanding outcomes of major system change. The key relationships are summarised in Fig. 4.

### Factors influencing implementation approaches

Implementation approaches differed across the two regions according to the degree to which implementation was phased, the degree to which implementation was linked to standards set out in service specifications and financial incentives, and the degree to which networks provided hands-on facilitation (Fig. 4, C3).

#### Degree to which implementation was phased (‘big bang’ vs ‘phased’)

London applied a ‘big bang’ approach, with a single ‘launch’ date for the whole system, from which time all suspected stroke patients were to be transferred to HASU, regardless of whether they were eligible for thrombolysis [32]. The single launch date grew from the view that the changes had to be pan-London, in order to ensure system-wide clarity about the model:

“if we started having north east London going off in one direction about some particular aspects of care and south east doing something a bit different then you very quickly lose the coherence” (stroke physician, London).

The launch was postponed by several weeks, to ensure all services developed adequate capacity to launch simultaneously, meaning all potential stroke patients in London could be taken to HASUs (London Network Board minutes, January–July 2010).

The London Stroke Project Board recognised that the ambulance service was central to agreeing the launch date:

“The Chair closed the discussion stating that the timing of the opening of HASUs needs to be agreed with London Ambulance Service.” (Minutes, Extraordinary Stroke Project Board meeting, June 2009).

In Manchester, service development took place over multiple phases. Changes to the referral pathway were piloted around one HASU as it developed, for example in terms of thrombolysis and repatriation processes. The referral pathway then altered several times over the course of implementation, as the remaining HASUs were launched and gradually extended their catchment areas (Stroke Project Board minutes, December 2008–April 2010). The network was aware of these changes, but they did not coordinate them, instead communicating with ambulance services to ensure their awareness of each change. This phased approach reflected a desire to minimise risk to vulnerable patients by ensuring that the referral pathway worked before scaling up to cover the whole system:

“…you could become completely overwhelmed and the whole thing might just collapse” (stroke physician, Manchester).

#### Use of service specifications and financial incentives

In both regions, service specifications were developed by local clinicians, and defined appropriate staffing, infrastructure, education, training, and audit processes. However, the London specifications quantified in greater detail how these services should be delivered (e.g. by identifying the number of specialist nursing and therapy staff required at different times of the day). While standards were used in service selection processes in both regions, only in London did the launch of services depend on these standards being achieved, assessed through a formal accreditation process.

In London, standards were linked to financial incentives, whereby receipt of enhanced funding for stroke services (the ‘stroke tariff’) was conditional on meeting these standards. Following the launch, services were required to meet additional standards reflecting further service developments (achieving a locally defined ‘gold standard’); subsequently, services were reviewed on an annual basis to assess whether standards continued to be achieved (if not, provisions were in place for the tariff to be ‘clawed back’). This approach gave change leaders in London a degree of control and assurance that services were likely to provide evidence-based care.

In Manchester, while commissioners endorsed the changes, payment was not associated with meeting standards on the basis that this might be seen as punitive and inconsistent with the collaborative approach employed. This meant that the new services could be launched whether or not standards had been met [32].

#### Degree of hands-on facilitation by networks

The networks played an important role in facilitating the changes. In both regions, they hosted regular meetings at which staff shared their learning from ongoing service development, for example in relation to developing thrombolysis pathways and managing transfer of patients from HASU to their local hospital. The focus on learning derived from the fact that the changes represented an attempt to standardise and integrate stroke care across what were, at the outset, relatively fragmented systems. The benefits of this were described by a member of the Manchester network:

“so much learning came out of it through this […] informing how the model should look and the paperwork, the communication protocols, the Standard Operating Procedures between, you know, it was all very emergent” (network representative, Manchester).

Significantly greater ‘hands-on’ facilitation was provided in London. This took the form of network staff project-managing and measuring service development throughout the implementation phase. Network staff engaged actively with senior hospital management and network leadership when implementation was not running to schedule, and in one case, a HASU lead was brought in to guide development of another HASU that was making limited progress. Network staff referred frequently to the pressures of service development, and their role in achieving it:

“The Programme Board was quite unrelenting really about, ‘these are the targets, we’ve got to hit them’” (network representative, London).

“We were there to remind them of what they had signed up to, to remind them of what they had committed to do and to remind them of the quality standards that they needed to meet but always in a supportive manner” (network representative, London).

This approach was driven by the tight timeline for a single launch date, linked to achieving service standards: this justified the network providing staff to carry out the intense facilitation approach.

In contrast, the Manchester changes had no explicit deadline by which all services had to achieve local standards, or to launch, and the network did not provide dedicated project management support. Overall, these characteristics indicate that implementation in Manchester was less actively facilitated by the network, reflecting their view of implementation as a collaborative endeavour, to be led by the services themselves:

“I don’t know whether it was an unwritten principle, it probably wasn’t a written principle but actually what we do is hold consensus and try and deliver this through unanimity” (commissioner representative, Manchester).

### Factors influencing implementation outcomes

As previously established (Fig. 3), there were differences, firstly, in implementation outcomes i.e. greater fidelity to the referral pathway in London than in Manchester and secondly, in intervention outcomes i.e. greater likelihood of providing evidence-based care in London than in Manchester (with provision equally high in London and Manchester HASUs, but lower in Manchester DSCs) [29]. We first discuss factors influencing fidelity to the referral pathway (model complexity, ‘big bang’ vs phased implementation, and degree of ‘hands-on’ facilitation by networks). Second, we discuss factors influencing service development (use of service specifications and ‘hands-on’ facilitation). As set out in the preceding section, many of the factors influencing implementation outcomes related to implementation approaches, including the degree to which implementation was phased, use of standards and financial incentives, and degree of hands-on facilitation (Fig. 4, C4).

#### Fidelity to referral pathway

Fidelity to the referral pathway was influenced strongly by how consistently it was understood by healthcare staff. Understanding of the referral pathway was influenced by the complexity of the models (number of decisions relating to patient transfer), and the number of phases in which these models were implemented.

#### Influence of model complexity on fidelity to referral pathway

The models implemented in London and Manchester differed in complexity. In both regions, the majority of stroke patients were transferred to hospital by ambulance. Ambulance representatives in both regions were consistent in making clear their preference for a simple model:

“we cannot give crews fragmented messages, you can’t say that you can get this type of care between 8 and 5 Monday to Friday but not on the second Wednesday of the month because there’s a meeting, crews don’t work that way” (ambulance service, London).

The difference between the 24 h pathway in London and the 4 h pathway in Manchester resulted in additional decision-making for ambulance crews in Manchester. As well as deciding on stroke diagnosis, they had to consider time of onset, and whether it would be possible to transfer the patient to HASU within 4 h of onset. As a result, a patient’s destination for care depended on potentially uncertain information:

“We need to have a definite time of onset […] or the time when they were last seen well, and if that time exceeds the four hours then we won't be taking them to the Hyper Acute Stroke Unit.” (ambulance service, Manchester).

While all London HASUs admitted patients 24/7, two of the three Manchester HASUs only operated an in-hours service. As noted by a representative of the ambulance services in Manchester, this may have made it more difficult to know where to take patients:

“Time’s always a challenge: between that time and that time they’ll go there, all the rest of the time they’ll go somewhere else. And that’s… that’s never, never easy to communicate or for people to remember” (ambulance service, Manchester).

In addition, some hospital staff indicated uncertainty about the Manchester referral model overall:

“I don’t understand who’s supposed to be going here and who’s supposed to be going there, and if I don’t, I bet other people don’t know.” (stroke physician, Manchester).

#### Influence of ‘big bang’ vs phased implementation on fidelity to the referral pathway

Ambulance staff indicated a strong preference for the ‘big bang’ approach employed in London:

“The one thing that we really did push for was a ‘go live’ date, not a ‘go live’ date in one area and another in other areas” (ambulance service, London).

Ambulance staff in Manchester suggested that the many changes made to the referral pathway over the course of implementation may have contributed to uncertainty, and thus limited fidelity to the referral pathway:

“If you phase it, it does create a degree of confusion. Because you start off with something, and then you change it, and then you change it, and then you may change it again” (ambulance service, Manchester).

#### Influence of ‘hands-on’ facilitation by networks on fidelity to referral pathway

The ‘hands-on’ facilitation provided by networks in London supported fidelity to the referral pathway. A key example was provision of training for ambulance staff to ensure clear understanding of the pathway:

“It’s not just the people on the road that need to understand that, it’s people in the control room as well, so they’re familiar. […] So there’s the protocol and then there’s the training to support that” (ambulance service, London).

Another task in pursuing fidelity to the referral pathway in London was ensuring that patients were not treated in hospitals no longer providing stroke care. A hospital where stroke services had been decommissioned had been continuing to receive stroke patients. To address this, a meeting with staff was organised:

“Somebody from the Stroke Network came to speak about ‘…we are not meant to be treating any stroke,’ […] So if you are here and you develop a stroke, your thing is to get you to [*local HASU*], rather than as I said, ‘We’re going to go and scan you first’. […] As a consequence of that, they’ve all gone…” (senior management, decommissioned service, London).

In Manchester, audit data indicated that a significant proportion of patients eligible for treatment in HASU were not being treated in one, reflecting concerns raised by clinical leads in oversight meetings (meeting minutes, 2009–2010). At the time of the Manchester 12 month review of the centralised system, it was noted that the network was working with both hospital and ambulance and hospital staff to corroborate data and identify potential solutions [38].

#### Service development processes

Reflecting the extent to which implementation was actively managed overall, service development in London and Manchester was influenced both by the degree to which service specifications and financial incentives were used, and the degree to which facilitation of service development was ‘hands-on’.

#### Influence of service specifications and financial incentives on service development

The London specifications presented standards that made clear what services had to provide, while the requirement to meet these standards was seen as an important driver for senior management to support these services:

“In some respect in terms of staff and sort of thing, it was taken out of our hands because the standards just lay it down, this is what you need for X number of beds” (HASU physiotherapist, London).

“Having to meet all these standards for assessment, it’s been a real driver for change and improvement. I think the reconfiguration has provided a stick for hospital management to invest in stroke services” (SU stroke physician, London).

In Manchester, standards were not linked to financial incentives, nor used as a criterion for the launch of services; this may have contributed to DSCs not providing the planned level of evidence-based care.

#### Influence of ‘hands-on’ facilitation by networks on service development

As services developed in London, the network’s ‘hands-on’ approach to facilitation was perceived by local staff as valuable in addressing difficulties:

“When we had problems, they [*the network*] wanted us to call them and say, ‘You know what, we’re a bit stuck here, what can you do to help?’ […] ‘is there experience you have from another site that might be helpful?’. I think we developed a very good relationship with them, and that was obviously key to, you know, opening the HASU” (HASU service manager, London).

Further, the ‘hands-on’ approach to facilitation influenced the timing of London’s ‘big bang’ launch. For example, it was only through this ongoing local engagement—and responsiveness to progress that was being reported—that the initial timescale for a coordinated launch was altered.

In Manchester, the networks facilitated learning across services, but did not provide staff to support service development, which may also have influenced provision of care in DSCs. This may have derived from the perception of comparatively limited resources dedicated to the Manchester centralisation:

“I heard that they [*London*] have £2.50 spent for every £1 spent in Manchester. As I say I don’t know if that’s accurate but it would seem that the financial thing wasn’t as such a consideration in London […] but it was a factor in Manchester” (network representative, Manchester)

### Understanding outcomes of major system change

In this section, we bring together the current findings with those from previous analyses to illustrate how components of major system change (Fig. 1) contributed to the significantly different outcomes associated with the changes to acute stroke services in Manchester and London. These relationships are summarised in Fig. 4, and described below.

The changes in London and Manchester appeared to be influenced significantly by the degree to which change leaders ‘held the line’ on the models to be implemented (Fig. 4, C1 and C2) [32]. The models implemented and implementation approaches employed played an important role in the implementation outcomes observed.

London’s inclusive 24 h model (i.e. all suspected stroke patients were eligible for HASU), requiring relatively few referral decisions to be made, increased likelihood of staff following the referral pathway. In contrast, Manchester’s 4 h model was significantly more selective (limiting the number of patients who were transferred to HASU) and complex, increasing uncertainty amongst staff about where suspected stroke patients were to be treated (C2). Further, these models were implemented differently, reflecting a contrast in the degree to which implementation was actively managed in the two regions (C3). London adopted a ‘big bang’ approach; the new system was launched on a single date, increasing likelihood of the referral pathway being followed. This launch was dependent on services being accredited against standards linked to financial incentives, increasing the likelihood of services providing evidence-based care. Significant ‘hands-on’ facilitation was provided by the London network to ensure that services met the required standards. In Manchester, services were launched in multiple phases, limiting confidence in the referral pathway. Service specifications were not linked either to service launch or financial incentives; this may in part have limited development of DSCs.

Implementation outcomes (C4) had a significant influence on intervention outcomes (C5). Almost all London patients were treated in a HASU, and all HASUs were likely to provide evidence-based care; this meant London patients were overall more likely to receive evidence-based care, and in turn had a larger reduction in mortality than patients in Manchester and elsewhere in England. In contrast, Manchester patients were far less likely to be treated in a HASU, with two thirds treated in DSCs, which were significantly less likely to provide evidence-based care; as a result, Manchester patients’ likelihood of receiving evidence-based care, and associated mortality, did not differ significantly from elsewhere in England [28, 29]. The 12 month review in Manchester noted national audit data indicating that DSCs were providing evidence-based care less frequently than HASUs [38]. Based on this information, and discussion with an external advisory group, it was agreed that further centralisation of acute stroke services should be explored [32].

## Discussion

This paper examines the complex, non-linear relationships between type of model selected, implementation approaches, implementation outcomes, and intervention outcomes. By analysing centralisation of acute stroke care in two regions, distinguishing between implementation outcomes and intervention outcomes (following Proctor [4]), we make a significant contribution to understanding of major system change [8], specifically in terms of the factors influencing outcomes [14]. We have demonstrated a number of inter-related factors potentially influencing such outcomes (as detailed in our previous research [28, 29]). Certain characteristics of intervention and implementation approaches are associated with more positive implementation outcomes and intervention outcomes, and many of these reflect existing implementation and diffusion theories, described below [1, 2, 4, 6–11].

In terms of intervention characteristics, we found that in this case ‘simpler’ and more inclusive referral pathways (such as London’s 24 h model) were more likely to be understood and followed by both hospital and ambulance staff. This effect might reflect such established concepts as ‘feasibility’ [4, 11], ‘compatibility’ [7], and ‘complexity’ [7, 10], whereby an intervention is more likely to be adopted if it is readily incorporated into existing or standard activities.

The concept of ‘execution’, i.e. where implementation is achieved as intended [10], was highly relevant to this analysis. In terms of timeliness of implementation, the advantages of a ‘big bang’ launch and associated planning, and the disadvantages of phased implementation, were clear: a single launch date gave clear understanding across all stakeholders when implementation was complete. This finding may seem counter-intuitive, given previous research indicating risks related to ‘big bang’ implementation [39]. However, in changes like these, where service models have multiple interdependent components, a ‘big bang’ approach appears to be beneficial, and represents an example of ‘adaptation’ [1, 9], where an implementation approach is selected to reflect the scale of the task and the complexity of the new system. Further research is required to establish the extent to which this finding applies to similar changes in different healthcare settings. Also associated with ‘execution’ was the use of standards: linking the launch of the new model to achieving standards appeared to increase the likelihood of there being uniform capacity to provide evidence-based care, but also gave a shared understanding of what had to be delivered in all services. By associating achievement of standards with financial incentives, the London changes reflected the hypothesised benefits of altering remuneration to encourage adoption of the intervention [9]. The contribution of ‘hands-on’ facilitation, for example by external change agents, to implementation outcomes, is acknowledged by various implementation frameworks [1, 10].

These differences in relation to ‘big bang’ vs phased launch, use of standards, and hands-on facilitation reflect an underlying contrast in the implementation approaches adopted in our studied regions, with implementation in London facilitated significantly more actively than in Manchester.

### Limitations

This paper has a number of limitations. First, because this research was retrospective in nature, interviewees were looking back on the changes, with some awareness (e.g. by monitoring national audit data) of the degree to which changes implemented had succeeded in influencing provision of evidence-based care. This may have affected the way in which they articulated their views of the implementation of the changes. Future research on changes of this kind would benefit from being carried out contemporaneously with the changes, ideally from pre-implementation stage, and extending over a sufficient time period to allow formal evaluation of impact on intervention outcomes, such as patient mortality. Second, while we believe our data indicate that the identified implementation strategies played a significant role in the implementation outcomes observed, the relative contribution of each component cannot be established. Third, we sampled only a proportion of services in this study, and other factors may have been important to implementation in other services in the reconfigured systems. However, we believe that by conducting interviews at pan-regional level, and sharing findings with local stakeholders, our findings provide a strong representation of implementation in both regions. Finally, this is a study of major system change in one particular domain of acute care. Studies of major system change in other acute (and non-acute) care settings would be of value to aid identification of potentially generalisable lessons.

## Conclusions

This paper used a framework drawing on key features of change identified in existing implementation theory to analyse two examples of major system change in acute stroke care. We found that model selection (‘simplicity’ and inclusivity) and implementation approach (single launch date, prioritisation of standards and financial incentives, and hands-on facilitation) make significant contributions to implementation outcomes observed, and in turn intervention outcomes [28, 29].

We believe this paper demonstrates the value of considering the interdependencies between intervention, implementation approach, and outcomes when planning and evaluating major system change. However, the particular relationships identified in this analysis may vary according to the nature of the change being implemented. The framework described in this paper is likely to be strengthened through further use in evaluating major system changes conducted in other healthcare settings.

## Additional files

Additional file 1: Governance level interviews topic guide. (DOCX 19 kb)

Additional file 2: Service level interviews topic guide. (DOCX 20 kb)
