# Supplementary material for: Explaining outcomes in major system change: a qualitative study of implementing centralised acute stroke services in two large metropolitan regions in England
Source: Implement Sci. 2016 Jun 3;11:80. doi: 10.1186/s13012-016-0445-z (PMC4891887; doi:10.1186/s13012-016-0445-z)
Supplement: Supplementary file 1 — Governance level interviews topic guide. (DOCX 19 kb) [file 13012_2016_445_MOESM1_ESM.docx]

**Background - interviewee**

To begin, please tell me

- how are/were you involved in stroke services?
- how were you involved with the reconfiguration?

**Background to the reconfiguration**

- What were the catalysts or drivers for the reconfiguration?
- e.g. national policy and research
- local drivers and key players – organisations, individuals

**Governing the reconfiguration**

- What groups and individuals led and governed the reconfiguration?
- How were you involved? How much time did you dedicate to this? e.g. how often did you attend meetings or events? How long were they?
- How did these work - and work together? e.g. attendance, focus, inter and intra group interactions
- What was the overall timeline for the reconfiguration?

**Developing the proposal for change**

- How were you involved in developing the proposal for change? Time dedicated?
- What were the key influences on how the proposals developed? E.g. groups, individuals
- Who was consulted? How were they consulted? e.g. interviews, focus groups, events, surveys
- Obstacles/enablers – how were these addressed/used?

**Agreeing the reconfiguration model**

- How were you involved in developing the reconfiguration model? Time dedicated?
- What reconfiguration options were developed? What were the key influences? E.g. groups, individuals
- Who was consulted on these, and how?
- What did you think of the options?
- How was the final model decided upon?

**Implementing the model**

- How were you involved in bringing about the changes? Time dedicated?
- How were the changes brought about, e.g. in opening/closing services, building capacity?
- What groups and individuals were central to implementing the model? How did they work?
- How were local stakeholders kept up to date on progress of the reconfiguration? e.g. newsletters, events
- Obstacles/enablers – how were these addressed/used? What were the levers for change?

**Outcomes**

- What changes were brought about by the reconfiguration? (e.g. organisation, service delivery, partnership working, patient outcomes, costs)
- Do you think these changes will be sustained?
- How were they measured? What capacity was/is dedicated to collecting these data? Are these measures reliable?
- Do you think the changes were worthwhile? Do other stakeholders feel the same?
- Would these changes have happened anyway?

**Reflections**

- Is there anything you would have done differently, in retrospect?
- Have you any further comments?
