# Supplementary material for: Explaining outcomes in major system change: a qualitative study of implementing centralised acute stroke services in two large metropolitan regions in England
Source: Implement Sci. 2016 Jun 3;11:80. doi: 10.1186/s13012-016-0445-z (PMC4891887; doi:10.1186/s13012-016-0445-z)
Supplement: Supplementary file 2 — Service level interviews topic guide. (DOCX 20 kb) [file 13012_2016_445_MOESM2_ESM.docx]

**Background**

To begin, please tell me a little about your background

- What is your role in this unit? How long have you worked here?
- Where have you worked previously, and in what settings? How long have you been involved in stroke care?

**Overview of changes**

In this evaluation, we are interested in learning about how the reconfiguration of stroke services in London/Greater Manchester influenced the ways in which care is organised and provided. Thinking back, would you tell me briefly

- How did you hear about proposals to reconfigure stroke services across London/Greater Manchester? What did you think of them? How were you involved in the changes?
- How did you hear about proposals to change the services provided in this unit/organisation? What did you think of them? How were you involved in these changes?
- What were services here like, initially? What changes came about as a result of the reconfiguration?

**Developing the services**

Please tell me about the changes that happened with the reconfiguration.

***Examples (with specific issues in parentheses)***

- **Processes** (e.g. services and therapies/protocols/standard operating procedures): can you describe the changes that happened here, and what had to be done to support them?
- **Staffing** (numbers/rota): what was done in terms of staffing to support these changes?
- **New roles** (e.g. ‘stroke nursing’, or more specialised roles): Have any new roles been created when developing this service as part of reconfiguration, or have new roles developed over time?
- **Skills and training**: What kinds of training have you or your colleagues received to support your work in the new services?
- **Becoming part of a wider system**: how have things changed in terms of how your service interacts with other parts of the local health system?
  - Other parts of hospital (other stroke units, A&E, radiology), other hospitals/units/primary care/ambulance
- **Governance:** Have any groups been set up here to oversee and support the changes you’ve mentioned, or to support high quality care more generally (both within the service and across the whole system)?

***General follow ups***

- What was its purpose? Why was it important?
- What was the background to this? Whose idea was it, and who was involved in agreeing it? What factors influenced these changes/decisions?
- How was this developed? How did it work (e.g. how it was led, who was consulted, how was it planned)?
- How were you involved? How did people work together to develop and implement these changes?
- What factors made a difference when implementing it? Were there any problems? How were these addressed?
- What difference did this change make (to Provision of care? Patients and carer experience? Outcomes/cost? Culture- inter & intra professional working, communication across the whole system?)
- What did you think of this change? What do other people think? How were these changes measured, and how were these measures agreed?
- How do other people (colleagues/management/patients and carers) feel about the changes?
- Are there any further changes that you think would be helpful?

**Overall impact of changes**

Overall, in what ways do you think the reconfiguration made a difference to stroke services, here?

- EXAMPLES: Provision of care? Patients and carer experience? Outcomes/cost? Culture- inter & intra professional working, communication across the whole system?
- FOLLOW UPS: How are these changes measured? How do other people (colleagues/management/patients and carers) feel about the changes?
- What factors influenced the changes (obstacles/supports)?
- Can you give me an example of this?

**Reflections**

Is there anything else you’d like to say about how the changes to services worked?

- Overall, how would you sum up the impact of the reconfiguration on how services are provided here (and across London/Greater Manchester)?
- What have you learned from this experience?
- Are there other changes you would you like to see happen?
- Is there anything you’d like to do differently in the future?
